# Recent knowledge of NFATc4 in oncogenesis and cancer prognosis

**DOI:** 10.1186/s12935-022-02619-6

**Published:** 2022-06-13

**Authors:** Qiu-Hua Zhong, Si-Wei Zha, Andy T. Y. Lau, Yan-Ming Xu

**Affiliations:** Laboratory of Cancer Biology and Epigenetics, Department of Cell Biology and Genetics, Shantou University Medical College, 22 Xinling Road, Shantou, Guangdong 515041 People’s Republic of China

**Keywords:** NFATc4, Cancer, Proliferation, Invasion, Migration, Prognosis, Drug resistance

## Abstract

Nuclear factor of activated T-cells, cytoplasmic 4 (NFATc4), a transcription factor of NFAT family, which is activated by Ca^2+^/calcineurin signaling. Recently, it is reported that aberrantly activated NFATc4 participated and modulated in the initiation, proliferation, invasion, and metastasis of various cancers (including cancers of the lung, breast, ovary, cervix, skin, liver, pancreas, as well as glioma, primary myelofibrosis and acute myelocytic leukemia). In this review, we cover the latest knowledge on NFATc4 expression pattern, post-translational modification, epigenetic regulation, transcriptional activity regulation and its downstream targets. Furthermore, we perform database analysis to reveal the prognostic value of NFATc4 in various cancers and discuss the current unexplored areas of NFATc4 research. All in all, the result from these studies strongly suggest that NFATc4 has the potential as a molecular therapeutic target in multiple human cancer types.

## Introduction

The nuclear factor of activated T-cells (NFAT) consists of transcription factors that regulate the expression of proinflammatory cytokines and related genes during the immune response. NFAT proteins comprise of five members, NFAT1 (NFATp or NFATc2), NFAT2 (NFATc or NFATc1), NFAT3 (NFATc4), NFAT4 (NFATx or NFATc3), and NFAT5 (TonEBP) [1]. Although the NFAT proteins have been extensively investigated in the immune system, their roles in oncogenesis and cancer progression have been less studied relatively. Recent researches showed that NFAT proteins are involved in the initiation, progression, and the prognosis of cancer. The change of NFAT proteins expression level and their post-translational modification (PTM) have been detected in various human solid tumors and cell lines, as well as haematological malignancies.

NFATc4 is encoded by a gene located on human chromosome 14 at q12 and was firstly isolated using a Jurkat T-cells and human peripheral blood lymphocytes (PBLs) cDNA library in 1995 [2]. NFATc4 is involved in several physiological processes, including the development and function of the immune, cardiovascular, musculoskeletal, and nervous systems [3–6]. It is also reported that NFATc4 could take part in cell growth, cell migration, tumor metastasis, and patient survival recently.

Over the past decades, NFATc4 remains relatively less explored when comparing with other NFAT proteins, in which there are still a huge research potential. In this review, we would like to discuss the recent knowledge on the expression, PTM and epigenetic regulations of NFATc4, its interaction partners and downstream components, as well as its biological function in cancers and other diseases.

## NFATc4 structure and expression

**Fig. 1:**
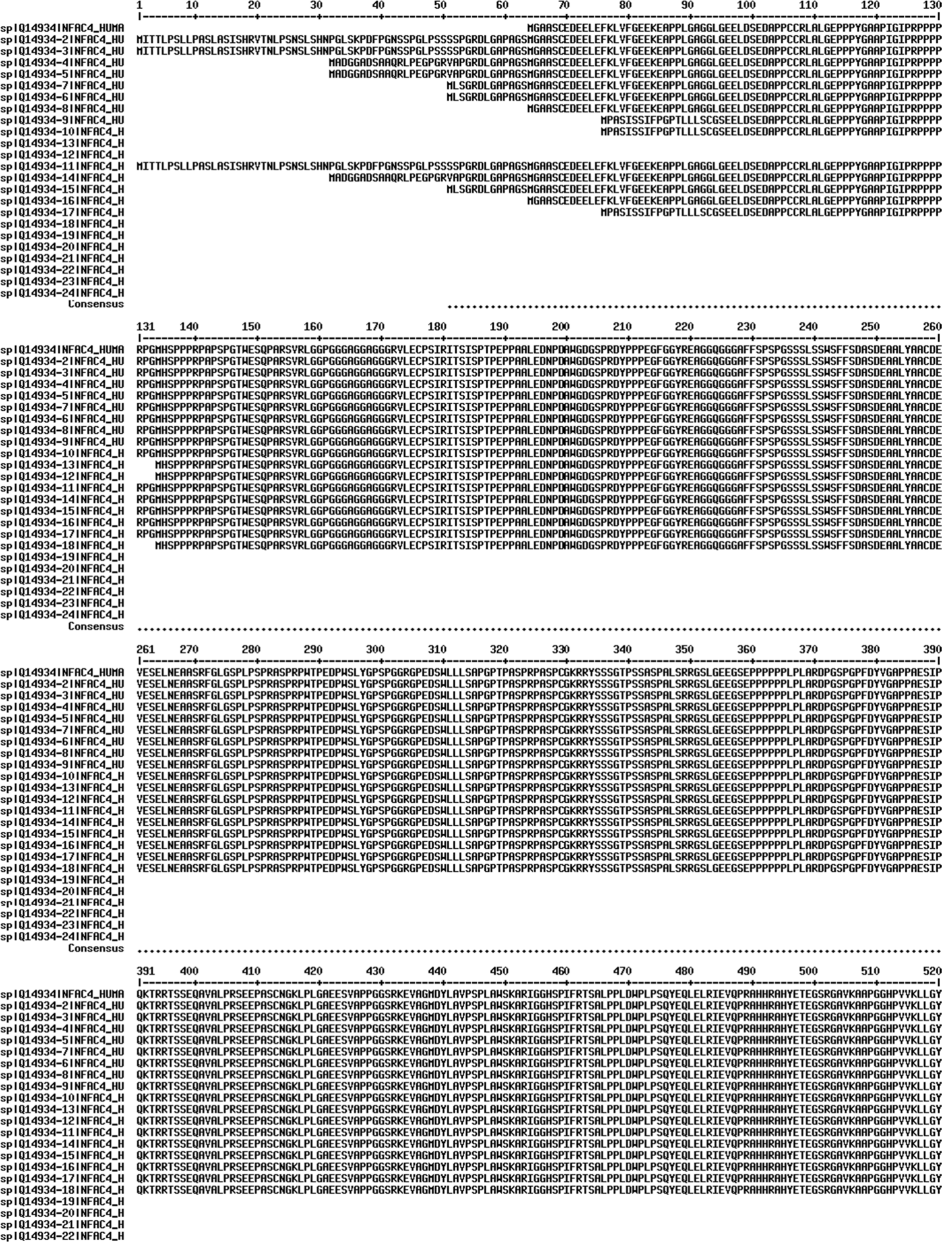

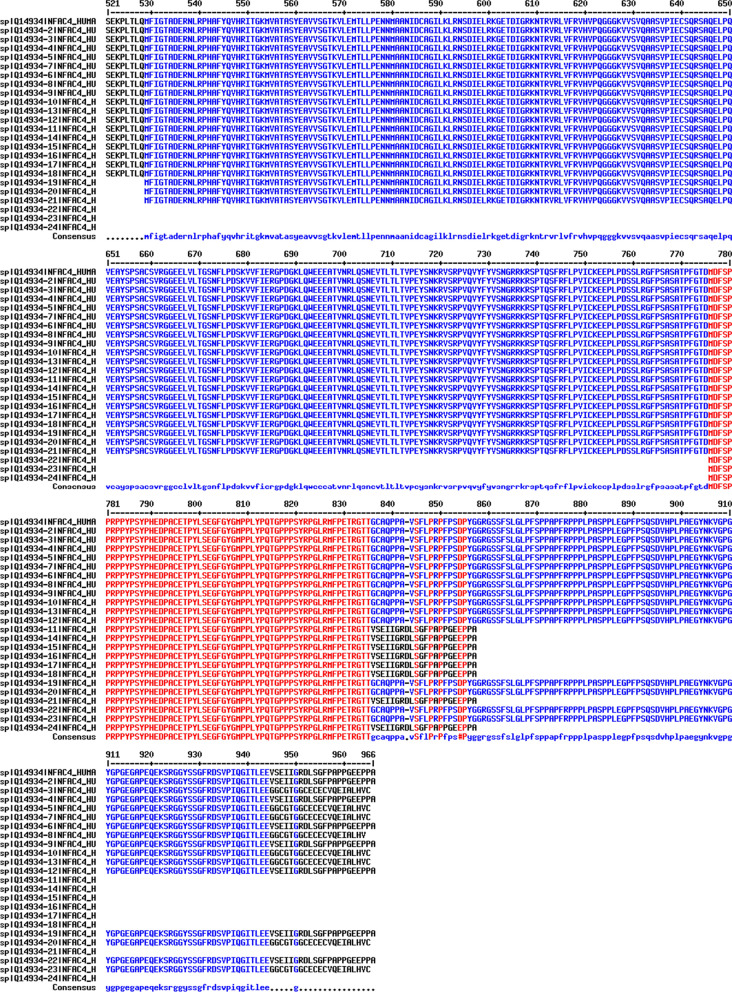
Amino acid sequence alignment of 24 human NFATc4 isoforms. Alignment was done by Multalin interface (http://multalin.toulouse.inra.fr/multalin/). Red color for high consensus (90%), blue color for low consensus (50%), and black color for unaligned residues. The symbol “#” for D, E, N, or Q. Dashes for optimal alignment

**Fig. 2:**
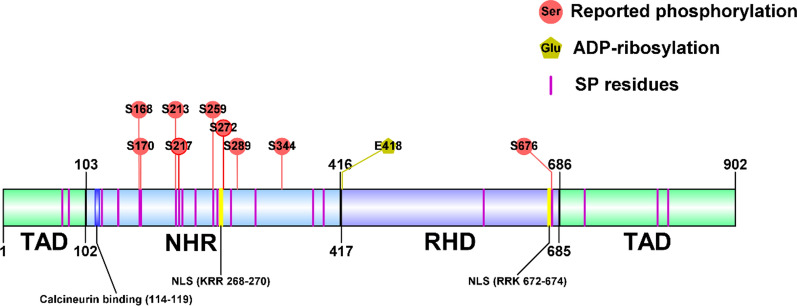
Schematic structure of NFATc4. The calcium-regulated NFATc4 protein composed of the NHR, the RHD and the N- and C-terminal transactivation domains. The NHR displays a calcineurin-binding site, a nuclear localization sequence (NLS). Another NLS is located in the RHD. Most of the NFATc4 serine-proline residues that are dephosphorylated upon calcineurin activation and phosphorylated by several kinases are located in NHR. The most conserved domain, RHD, also called DNA binding domain (DBD) that directly contacts with DNA is indicated as the DNA-binding loop. Amino acids marked with red circles indicate reported phosphorylation sites; amino acid marked with green pentagon indicates ADP-ribosylation site; purple lines indicate serine-proline residues. There are 21 serine-proline repeats in the NFATc4 amino acid sequence while 16 serine-proline repeats located in NHR and RHD

NFATc4 has 24 isoforms produced by alternative splicing (Table 1, Fig. 1). Among these isoforms, isoform 1 is regarded as the canonical sequence and all the reported researches are based on isoform 1. As the schematic structure shown (Fig. 2), NFATc4 contains a conserved Rel homology domain (RHD) which includes the DNA-binding motif, and two transactivation domains (TADs) which locate at the N-terminus and C-terminus respectively. Between the TAD in the N-terminus and the RHD is the regulatory domain (NHR) where most SP motifs located, and many biological functions of NFATc4 are mediated by the phosphorylation and dephoshporylation of the SP motifs. In addition, there are two nuclear localization signals located in the NHR and the RHD.

**Table 1:** Isoforms of NFATc4

Name	Also known as	Length (amino acids)	Mass (Da)	Identifier (Uniprot)
Isoform 1	ID-IXL	902	95,449	Q14934-1
Isoform 2	IA-IXL	965	101,680	Q14934-2
Isoform 3	IA-IXi	964	101,513	Q14934-3
Isoform 4	IC-IXL	934	98,436	Q14934-4
Isoform 5	IC-IXi	933	98,269	Q14934-5
Isoform 6	IB-IXL	915	96,662	Q14934-6
Isoform 7	IB-IXi	914	96,495	Q14934-7
Isoform 8	ID-IXi	901	95,282	Q14934-8
Isoform 9	IE-IXL	890	94,146	Q14934-9
Isoform 10	IE-IXi	889	93,978	Q14934-10
Isoform 11	IA-IXS	857	90,525	Q14934-11
Isoform 12	IEi-IXL	832	88,270	Q14934-12
Isoform 13	IEi-IXi	831	88,103	Q14934-13
Isoform 14	IC-IXS	826	87,282	Q14934-14
Isoform 15	IB-IXS	807	85,508	Q14934-15
Isoform 16	ID-IXS	794	84,294	Q14934-16
Isoform 17	IE-IXS	782	82,991	Q14934-17
Isoform 18	IEi-IXS	724	77,115	Q14934-18
Isoform 19	IV-IXL	437	47,373	Q14934-19
Isoform 20	IV-IXi	436	47,206	Q14934-20
Isoform 21	IV-IXS	329	36,219	Q14934-21
Isoform 22	VI-IXL	190	20,104	Q14934-22
Isoform 23	VI-IXi	189	19,937	Q14934-23
Isoform 24	VI-IXS	82	8,950	Q14934-24

**Fig. 3:**
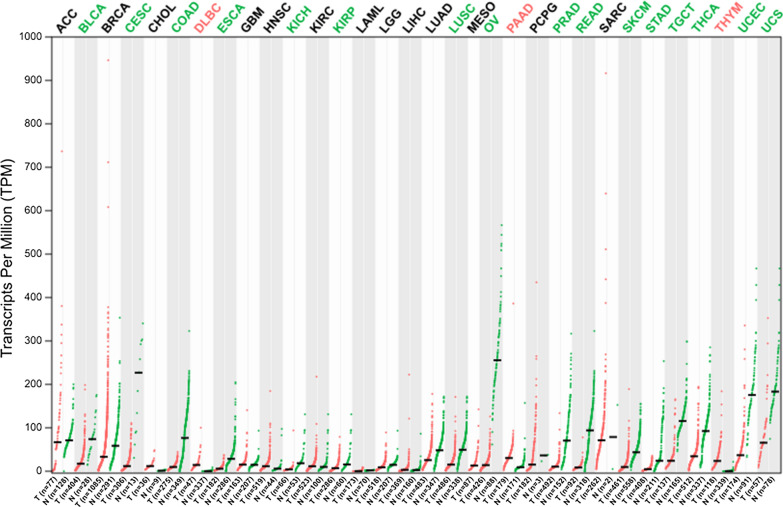
mRNA level analysis of NFATc4 in various cancers. The NFATc4 mRNA analysis are performed on matched TCGA tumor tissues and normal tissues (TCGA normal and GTEx data) using GEPIA (http://gepia.cancer-pku.cn/index.html). Cancers in green letters have lower NFATc4 expression; Cancers in red letters have higher NFATc4 expression. The NFATc4 mRNA is at a low level in 16 types of cancer, while it is at a high level in 3 types of cancer

At first, NFATc4 was reported to be rarely expressed in the immune system [2], which was different from other NFAT proteins. With the increasing researches of NFATc4, its expression in many tissues can be detected (Table 2) including the immune system. The reason of NFATc4 down-regulation in immune cells is the lack of expression of T-box transcription factor TBX5 [7]. Today, studies show that NFATc4 played important roles in cardiovascular, muscular, skeletal, and nervous systems. Also, NFATc4 is involved in oncogenesis and some immune-related conditions like asthma [8], graft rejection [9], colitis [10] and osteoarthritis [11]. In TCGA and GTEx database, 16 types of cancer show lower mRNA levels of NFATc4 when comparing with normal tissues, while 3 types of cancer show higher mRNA levels (Fig. 3). Yet, there are only a few researches cover the protein level of NFATc4 in cancer and normal tissues.

**Table 2:** The expression of NFATc4 in different tissues or cell lines

Tissue/cell line	Expression level	References
Non-small cell lung cancer tissue (Human)	Protein	[12]
Breast cancer cell lines (T47D, ZR-75-1) (Human)	Protein	[13]
Breast cancer patients’ PBMC (Human)	mRNA	[14]
Ovarian cancer cell lines (HeyA8, SKOV3) (Human)	Protein	[15, 16]
Cervical cancerous cell lines (C33A, HeLa, SiHa) (Human)	Protein	[17]
Skin cancer tissue and cell lines (Human)	Protein	[18, 19]
Pancreatic cancer tissue (Mouse)	Protein	[20]
Colon cancer cell line HT29 (Human)	Protein	[21]
Glioblastoma (Human)	Protein	[22]
Schwannoma (Human)	Protein	[23]
Lung (Mouse)	Protein	[24]
Spleen (Mouse)	Protein	[24]
Liver (Mouse)	Protein	[24]
Regulatory T cells (Tregs) (Human)	mRNA	[3]
Bone marrow-derived macrophages (Mouse)	Protein	[25]
Cardiomyocytes (Human)	Protein	[26]
Cardiomyocytes (Mouse)	Protein	[4]
Keratinocytes (Human)	Protein	[27]
Adipocytes (Mouse)	Protein	[28]
Primary hippocampal neuron (Rat)	Protein	[6]
Skeletal muscles (Rat)	Protein	[5]

## Modulation of NFATc4 activation

### Epigenetics

Epigenetics is the study of heritable changes in gene expression without alteration of the underlying DNA sequence that are mediated by epigenetic factors like methylation of DNA, modification of histone and non-coding RNA regulation [29]. It is reported that NFATc4 protein expression can be suppressed by several microRNA: miR-34a-5p [30], miR-29a-3p [31], miR-133a [32], miR-182 [14], miR-145-5p [33], miR-7134-5p [34], and miR-140 [11]. However, DNA and histone modification haven’t been explored yet.

### Post-translational modification

At the resting state, a balance between the tonic activity of calcineurin phosphatase and the protein kinase is required to maintain cytosolic localization of NFATc4 [35]. Upon an increase of intracellular calcium, activated calcineurin dephosphorylates NFATc4 and promotes its nuclear translocation. Transcription termination promotes rephosphorylation of NFATc4, then NFATc4 can be translocated to the cytosol. Except phosphorylation and dephosphorylation, NFATc4 can also be subjected to ADP-ribosylation, ubiquitination, and deacetylation (Table 3). Specifically, ADP-ribosylation at NFATc4 DNA binding domain could augment the transcriptional activity of NFATc4 [36] whereas ubiquitination of NFATc4 promoted NFATc4 degradation and thus suppressed its transcriptional activity [37]. Also, it has been shown that the deacetylation of NFATc4 by SIRT6 could suppress NFATc4 dephosphorylation [38].

**Table 3:** Post-translational modification (PTM) of NFATc4

Enzyme	PTM	Studied sites	Verification	References
Calcineurin	Dephosphorylation	multisites	3	[2]
p38 MAP kinase	Phosphorylation	Ser168, 170	3, 4	[35, 39]
mTOR	Phosphorylation	Ser168, 170	2, 3	[35]
MAPK7 (ERK5)	Phosphorylation	Ser168, 170	2, 3	[35]
IRAK-1	Phosphorylation	Ser168, 170	3	[40]
JNK1/2	Phosphorylation	Ser213, 217	2, 3, 4	[41]
CDK3	Phosphorylation	Ser259	2, 3, 4	[19]
PKA	Phosphorylation	Ser272, 289	2, 3, 4	[42]
RSK2	Phosphorylation	Ser289, 344, 676	1, 2, 3, 4	[43, 44]
PARP-1	ADP-ribosylation	Glu418	1, 2, 3, 4	[36]
-	Ubiquitination	-	3	[37]
SIRT6	Deacetylation	-	3	[38]

1: Mass spectrometry; 2: Autoradiography; 3: Immunoblot; 4: Site mutation

### Interaction proteins

Besides post-translational modification, NFATc4 transcriptional activity is also modulated by the interaction proteins (Table 4).

**Table 4:** Interaction proteins of NFATc4

Protein	Co-activator/ Co-repressor	Downstream genes	References
AP-1	Co-activator	*IL-2, VEGF*	[2, 45]
CBP	Co-activator	*TNFα*	[46]
ERα	Co-repressor	*LCN2*	[13]
ERα/β	Co-repressor	*IL-2*	[47]
ERα/β	Co-activator	*pS2, cathepsin D*	[48]
GATA4	Co-activator	*BNP, CnA*β	[49, 50]
Lipin-1	Co-repressor	*TNFα, FABP4*	[51]
PARP-1	Co-repressor	*BNP*	[36]
PGC-1α	Co-repressor	*-*	[52]
SOX10	Co-activator	*P0*	[53]
14-3-3	Co-repressor	*ANP*	[42, 54]

## NFATc4 functions in cancer

### Lung cancer

**Fig. 4:**
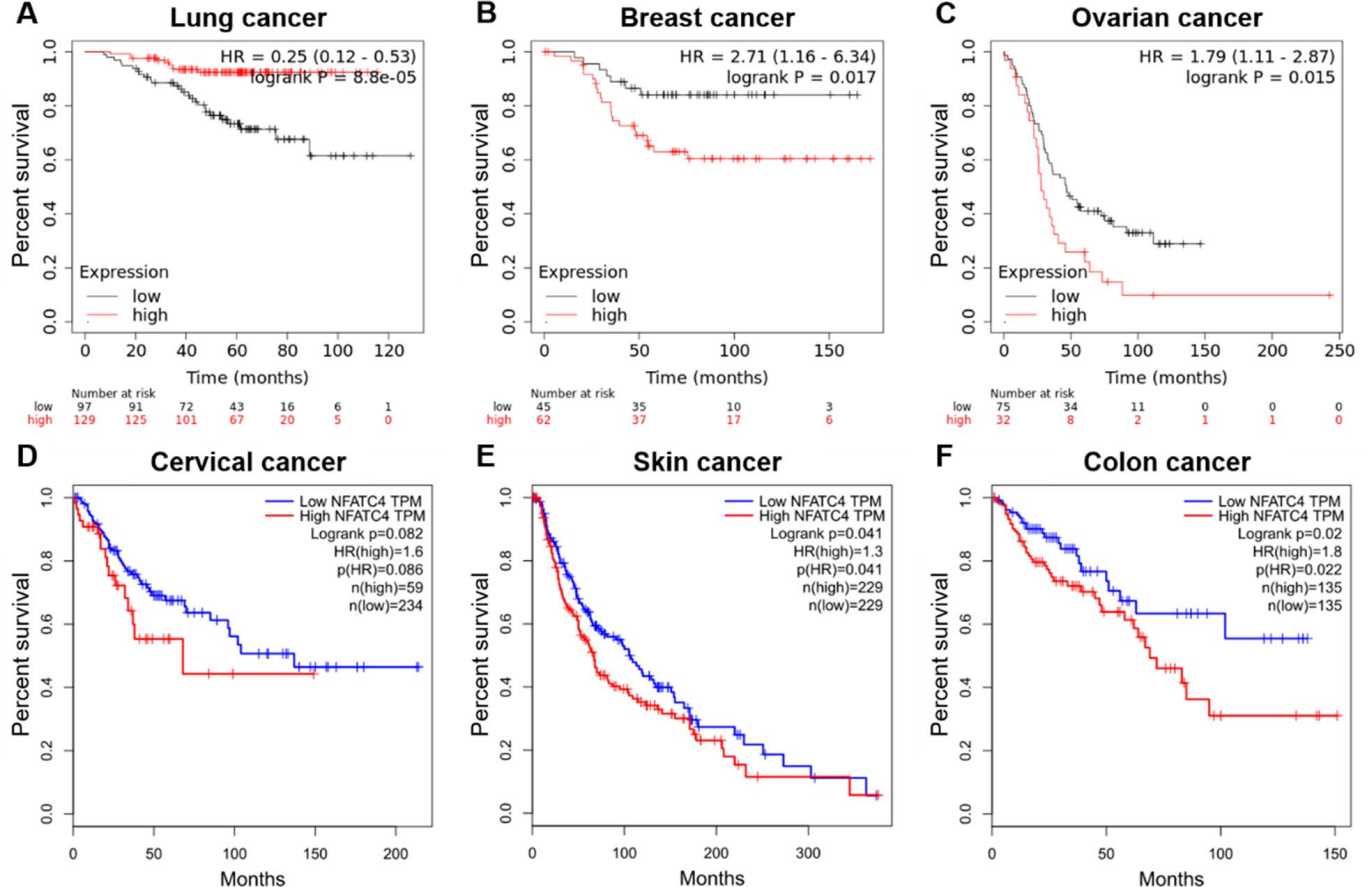
Survival analysis of NFATc4 in various cancers. **A**–**C** The data is from GEO database and analyzed by KM plotter (https://kmplot.com/analysis/index.php?p=background). **A** The GSE31210, a lung cancer dataset, shows that the high expression of NFATc4 is significantly related to favorable prognosis. **B** The GSE12276, a breast cancer dataset, shows that the high expression of NFATc4 is significantly related to poor prognosis. **C** The GSE26193, a ovarian cancer dataset, shows that the high expression of NFATc4 is significantly related to poor prognosis. **D–F** The data is from TCGA database and analyzed by GEPIA (http://gepia.cancer-pku.cn/index.html). **D** The CESC, a cervical cancer dataset, shows that the high expression of NFATc4 is significantly related to poor prognosis. **E** The SKCM, a skin cancer dataset, shows that the high expression of NFATc4 is significantly related to poor prognosis. **F** The COAD, a colon cancer dataset, shows that the high expression of NFATc4 is significantly related to poor prognosis

Lung cancer is the leading cause of cancer induced death worldwide. The past two decades of research have yielded encouraging results about the disease mechanism and therapy of lung cancer. Nonetheless, the need of new molecular targets remain to be intensively elucidated to prompt the development of diagnosis and new therapies. Chen’s research revealed that the NFATc4 positive rate is 28.3% in tumor tissues and 1.3% in adjacent normal tissues from 159 non-small cell lung cancer patients [12]. They found a positive correlation between the expressions of NFATc4 and COX-2 which were both overexpressed in non-small cell lung carcinoma. In addition, NFATc4 transcriptionally regulated COX-2 expression and prevented human bronchial epithelial cells BEAS-2B from apoptosis caused by arsenite and vanadium treatment [55, 56]. Another research reported that knockdown of NFATc4 expression resulted in a radioprotective effect on A549 lung cancer cell line [57]. The survival analysis of GSE31210 dataset from GEO database indicates that high NFATc4 level tends to a favorable prognosis (Fig. 4A).

Although the research of NFATc4 in lung carcinoma is a bit less, NFATc4 has presented relatively high expression in lung cancer and involved in radiotherapy and the prognosis.

### Breast cancer

Accumulated evidences have indicated that NFATc4 plays essential roles in regulating the proliferation, invasion and migration of breast cancer cells. It has been shown that the expression of NFATc4 can be detected in breast cancer cells and a subset of breast cancer patients. NFATc4 is overexpressed in breast cancer with 65% positive in cancerous tissue and 15% positive in noncancerous tissue. Notably, it was found that NFATc4 not only interacted with the ligand-independent transactivation domain of ER (ERα and ERβ) but also increased its binding to the estrogen-responsive elements resulting in the up-regulation of pS2 and cathepsin D expression. Meanwhile, knockdown of NFATc4 reduced the cell growth rate in ZR-75-1 cells which showed high NFATc4 expression level [48].

Tumor invasion and metastasis may lead to cancer progression and poor outcomes. NFATc4 also shows potential regulation of these aspects in breast cancer cells. Interestingly, NFATc4 expression is restricted to ERα positive cell lines at both transcriptional and translational levels. Remarkably, NFATc4 inhibited cell invasion and migration through regulating actin redistribution. Furthermore, research identified that NFATc4/ERα decreased cell migration capacity through transcriptionally repression of LCN2 expression [13]. Meanwhile, overexpression of ERα promoted the nuclear translocation of NFATc4. NFATc4/ERα and NFATc4/ERβ inhibited the transcriptional activity of IL-2 in breast cancer cells, especially ERα. Phosphorylation of ERα at different sites affected the regulation of ERα on NFATc4 transcriptional activity [47]. Additionally, NFATc4 is highly co-expressed with androgen receptor (AR) which is expressed in 88% ER-positive breast tumors [58].

Extracellular vesicles (EVs) are kinds of two-layer secreted vesicles which play pivotal roles in mediating cell signal transduction and pathological process. The tumor-derived EVs are thought as the promising therapeutic approaches through mediating intercellular communication in the tumor microenvironment. EVs produced by breast cancer cells expressing NFATc4 inhibited cell invasion effectively among different types of cancer cell lines (triple negative breast cancer, invasive melanoma, glioblastoma, and pancreatic cancer). Intra-tumor EVs injection presented a significant inhibition of the tumor volume and metastasis in tumor-bearing mice. To further explain EVs function, de Camargo et al. demonstrated EVs from the NFATc4-expressing cells decreased the cell proliferation rate and induced cell apoptosis through macrophages recruitment in the 2D cell culture system [59]. In addition, NFATc4 down-regulated during (E)-4-chloro-2-((3-ethoxy-2-hydroxybenzylidene) amino) phenol (ACES) induced apoptosis through p38-β/SAPK in MCF-7 breast cancer cells, indicating that NFATc4 could be a latent cancer therapeutic target [60].

Based on survival analysis, NFATc4 is an unfavorable prognostic factor in breast cancer (Fig. 4B). Thus, these observations collectively highlighted the critical role of NFATc4 in breast cancer development. On the one hand, NFATc4 could interact with ER and transcriptionally regulate downstream genes, and promote the cell growth. On the another hand, NFATc4 presenting high expression at mRNA and protein levels in ERα-positive cells repressed LCN2 and led to inhibition of invasion and migration capacities of breast cancer cells. NFATc4 could also play an important role in tumor immune microenvironment through EVs from NFATc4-expressing cells.

### Ovarian cancer

Ovarian cancer ranks the fifth cancer-induced death among women. Patients diagnosed with ovarian cancer are treated with complete resection surgery and neoadjuvant or adjuvant chemotherapy. Recently, it is considered that many patients were overtreated especially those at early stage. It is important to develop the molecular signature to indicate more suitable individual therapeutic strategy. Accumulating studies identified that NFATc4 is involved in the ovarian cancer, too. NFATc4 is overexpressed in ovarian cell lines 3D spheroids comparing with adherent cells [16]. In ovarian quiescent cancer stem-like cells (CSCs), NFATc4 is also overexpressed [15]. NFATc4 directly bound to the CXCR4 promoter at the DNA sequence of -114 to + 59 relative to the transcriptional start site and correspondingly increased CXCR4 expression. When the HeyA8 ovarian cell line 3D spheroids were treated with Cyclosporine A (a usual NFAT inhibitor), the cells dissociated from the 3D spheroids. CXCR4 also presented as a key driver in tumor progression in HeyA8 xenograft model. Moreover, phospho-ERK participated in modulating CXCR4 expression through inhibiting NFAT transactivation [16]. Interestingly, NFATc4 is enriched in ovarian quiescent CSCs and inhibited the cell growth through arresting cells at G0 phase. It was found that NFATc4-involved quiescence could play a role in cisplatin resistance. Cisplatin treatment activated NFATc4 and promoted NFATc4 translocation into the nucleus in multiple ovarian cell lines. Remarkably, MYC overexpression may partially inhibit NFATc4-regulated quiescence, which indicated a potential therapy for cisplatin resistance patients [15]. Besides, NFATc4 acted as a risk factor in the survival of ovarian cancer analyzed from the GSE26193 dataset (Fig. 4C).

As a transcriptional factor, activated NFATc4 took part in ovarian cancer progression in various regulatory ways. NFATc4 could promote tumor formation through CXCR4 expression up-regulation whereas NFATc4 inhibited cell proliferation by down-regulating MYC during cisplatin resistance-associated quiescence.

### Skin cancer

Skin-derived malignancies include squamous cell carcinoma, basal cell carcinoma, and malignant melanoma. These malignancies partially related to harmful factors exposure. NFATc4, a stress response transcriptional factor, was found overexpressing in many skin cancer cell lines comparing with HaCaT cells. Consistently, NFATc4 showed high protein level in various tissues of skin malignancies. The ectopic expression and activation of NFATc4 may markedly facilitate the progress of skin cancer. Overexpressing NFATc4 increased the cell proliferation, colony formation, migration, invasion, as well as tumor volume [19], while knockdown of NFATc4 by siRNA or inhibiting NFATc4 activity by tacrolimus (FK506) or ascomycin (FK520) suppressed skin cancer cell migration and invasion [18]. Further study elucidated that CDK3 phosphorylated NFATc4 at Ser259 and enhanced NFATc4 effect on skin cancer, and EGF treatment could promote CDK3/NFATc4-mediated cell transformation and proliferation. Of note, both CDK3 and NFATc4 are highly expressed among various types of skin tumor tissues compared with normal tissues [19]. Survival curve drawn with TCGA-SKCM indicates a poor outcome in patients with high NFATc4 level.

### Pancreatic cancer

Pancreatic ductal adenocarcinoma (PDAC) is one of the most aggressive malignances for its dramatically low 5-year survival rate. A recent study showed that NFATc4 is positively expressed in cell nucleus of human chronic pancreatitis and it is highly induced during pancreatic acinar-to-ductal metaplasia. NFATc4 could play a pivotal role in caerulein-induced pancreatic cancer initiation through NFATc4 transcriptional overexpression of SOX9 [20].

### Brain cancer

Since the blood-brain barrier and the crucial function of brain, malignant brain tumors remain a therapeutic challenge, in spite of surgical and medical advancement. NFATc4 was required and activated in doxorubicin-mediated cell death as well as doxorubicin-induced inhibition of  cell migration and invasion in glioblastoma cell lines [22]. In Schwannoma, NFATc4 and SOX10 synergistically enhanced KROX20 activity and induced P0 transcription to regulate the cell proliferation. When overexpressing SOX10 in Merlin-null Schwannoma cells in which the SOX10 level was reduced to restore Merlin tumor suppressor, NFATc4 was activated with nuclear translocation and the cell growth was alleviated [23, 53].

### Other cancers

Besides from the above mentioned, the involvement of NFATc4 in cancer was gradually discovered in some other types of cancer, including hepatic cell carcinoma, colon cancer, cervical cancer, and leukemia.

It is well-known that certain patients with non-alcoholic fatty liver disease, the most common chronic liver disorder, may also develop non-alcoholic steatohepatitis (NASH). NASH is a disease associated with hepatocyte injury and inflammation, which could then lead to liver fibrosis, and subsequently cirrhosis and/or hepatic cell carcinoma (HCC). NFATc4 was activated and translocated into nucleus during methionine-choline diet-induced NASH, while knockdown of NFATc4 inhibited both methionine-choline diet-induced NASH and obesity-related NASH in mice. NFATc4 directly bound to PPARα and interfered with lipid metabolism through negatively regulating PPARα transcriptional activity, and then contributed to the development of NASH [24, 61]. These findings provided a potential therapeutic target in preventing NASH progression through inhibiting NFATc4 expression and activation. Remarkably, it was found that the inhibition of calcineurin/NFATc4 signaling mediated by RCAN1.4 could retard liver fibrosis [62].

NFATc4 is also involved in colon cancer, another digestive system neoplasm. It has been reported that arsenic sulfide (As_4_S_4_) and cyclosporine A (CsA) synergistically decreased colon cancer cell proliferation through the regulatory PML and p53 mediated NFAT signal pathway. CsA is a well-known NFAT inhibitor, meanwhile, As_4_S_4_ could inhibit the transcriptional expression of NFATc1, NFATc3, and NFATc4, except NFATc2 [63].

Accumulating bioinformatical analyses about immune-related genes (IRGs) indicated that NFATc4 was recognized as the key immune gene to predict the poor prognosis in cervical cancer [64, 65] and acute myelocytic leukemia (AML) [66]. In AML, NFATc4, an immune-related transcriptional factor, was co-expressed with immune gene set of T cell co-stimulation and was involved in Tregs recruitment. Simultaneously, NFATc4 was co-expressed with ATP-binding cassette transporter signaling pathway [66]. In addition, NFATc4 played a role in primary myelofibrosis (PMF), a stem cell-derived clonal myeloproliferative neoplasm. In PMF megakaryocytic cells, NFATc4 was up-regulated by activation of FL/Flt3 axis, while the Flt3 inhibition reinforced the Flt3/p38-MAPK axis effect on PMF dysmegakaryopoiesis [67].

**Fig. 5:**
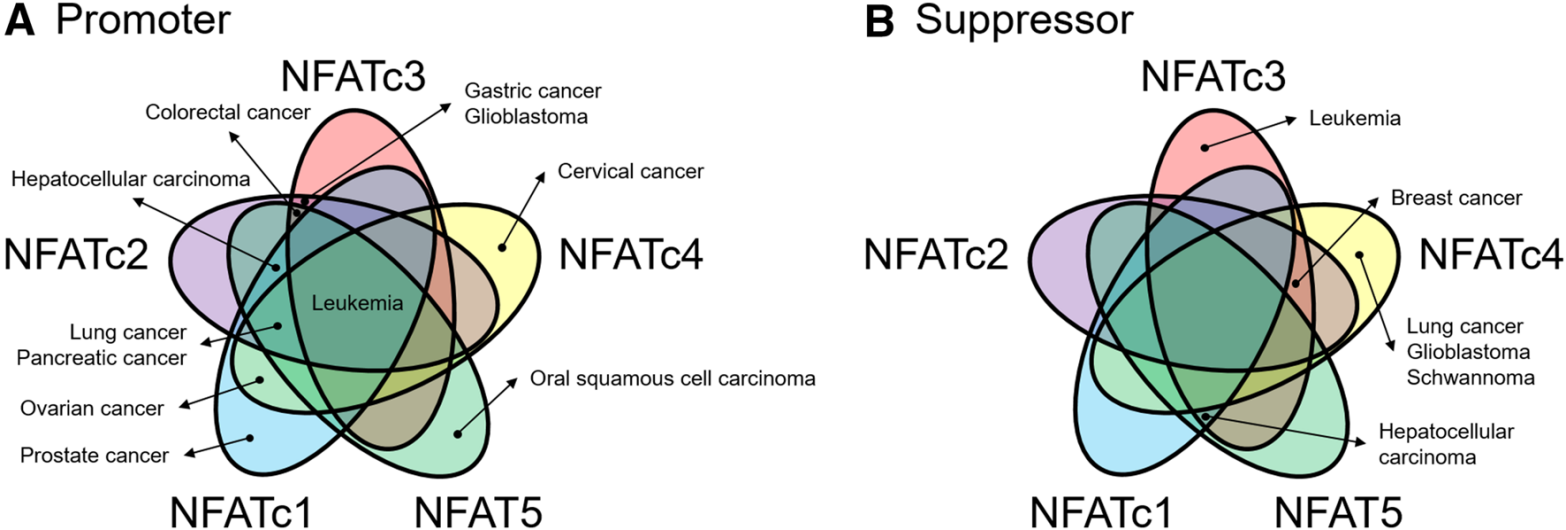
Venn diagrams displaying common and distinct cancer types associated with NFAT proteins; **A** NFAT proteins act as promoter; **B** NFAT proteins act as suppressor

In summary, these emerging observations in the past ten years emphasized the significant roles of NFATc4 in the progression of varieties of human malignancies (Table 5). NFATc4 may perform a dual-function during tumor progression like other NFAT proteins (Fig. 5). In the aspect of oncogenesis, NFATc4 activation promoted the EGFR-stimulated process of acinar-to-ductal metaplasia in pancreas and NFATc4 was activated in the NASH and liver fibrosis. NFATc4 also provided as a novel potential molecular target for cancer therapies, since NFATc4 participated in cell proliferation, migration, invasion, colony formation, 3D spheroid formation, and cell apoptosis. Furthermore, NFATc4 was related to the tumor immune microenvironment through recruiting macrophage and Tregs. Finally, the expression of NFATc4 could effectively evaluate the risk and outcome of the patients. Hence, the further study of NFATc4 function in various cancers could help to prevent the precancerous development and improve the individualized cancer therapy.

**Table 5:** Downstream genes of NFATc4 in cancer

Gene	NFATc4 activity	Related cancer	Regulation	References
*COX-2*	Promotes	Lung cancer	Cell apoptosis	[55, 56]
*TNFα*	Promotes	-	Cell apoptosis	[68]
*pS2*	Promotes	Breast cancer	Cell growth	[48]
*cathepsin D*	Promotes	Breast cancer	Cell growth	[48]
*LCN2*	Represses	Breast cancer	Cell migration	[13]
*CXCR4*	Promotes	Ovarian cancer	Tumor spheroid formation	[16]
*SOX9*	Promotes	Pancreatic cancer	Cancer initiation	[20]
*KROX20*	Activates	Schwannoma	Cell growth	[23, 53]
*P0*	Promotes	Schwannoma	Cell growth	[23, 53]

## NFATc4 functions in other diseases

In the past three decades, the researchers have mainly focused on the function of NFATc4 in cardiac hypertrophy, neurodegenerative disease, immune-related disease, and ischemia–reperfusion injury. In hypertrophic cardiomyocytes, overexpressed and activated NFATc4 transcriptionally up-regulated downstream genes like ANP and BNP [69]. In the cortex of Alzheimer’s disease patients, increased expression of NFATc4 and calcineurin were detected, and activation of NFATc4 regulated the downstream factor Aβ protein to modulate the neurodegeneration [70]. Furthermore, hepatic ischemia–reperfusion injury could be alleviated by pre-treatment with NFATc4 inhibitor FK506, while the Bcl-2/Bax ratio increased [71].

## Conclusions and future prospects

Taken together, growing evidences revealed the molecular regulatory mechanism of NFATc4 and its crucial role in the cancer development. Transcription activity of NFATc4 is mainly regulated by the calcineurin and phosphokinase, meanwhile acetylation could facilitate the dephosphorylation of NFATc4. Specifically, ADP-ribosylation at NFATc4 DNA binding domain could augment the transcriptional activity of NFATc4 whereas ubiquitination of NFATc4 promoted NFATc4 degradation and thus suppressed its transcriptional activity. According to recent decade of researches, NFATc4 protein has been recognized as an important transcription factor during the tumor initiation, cell growth, migration, invasion, metastasis, and drug resistance.

However, the regulation of NFATc4 is not deeply studied compared with other NFAT proteins. Since the activated NFATc4 has been detected in the precancerous lesions and cancers, its downstream genes are noteworthy to be elucidated.

Thus, there are compelling reasons to believe that it is necessary to further study the unrevealed mechanisms of NFATc4 in cancer. NFATc4 may be considered as a new target to prevent the malignant cancer progression.

## Data Availability

All data generated or analyzed during this study are included in this published article.

## Abbreviations

NFATc4: Nuclear factor of activated T-cells, cytoplasmic 4
PTM: Post-translational modification
RHD: Rel homology domain
TAD: Transactivation domain
EVs: Extracellular vesicles
PBMC: Peripheral blood mononuclear cell
NASH: Non-alcoholic steatohepatitis
HCC: Hepatic cell carcinoma
PMF: Primary myelofibrosis
AML: Acute myelocytic leukemia
ACC: Adrenocortical carcinoma
BLCA: Bladder urothelial carcinoma
BRCA: Breast invasive carcinoma
CESC: Cervical squamous cell carcinoma and endocervical adenocarcinoma
CHOL: Cholangio carcinoma
COAD: Colon adenocarcinoma
DLBC: Lymphoid neoplasm diffuse large B-cell lymphoma
ESCA: Esophageal carcinoma
GBM: Glioblastoma multiforme
HNSC: Head and neck squamous cell carcinoma
KICH: Kidney chromophobe
KIRC: Kidney renal clear cell carcinoma
KIRP: Kidney renal papillary cell carcinoma
LAML: Acute myeloid leukemia
LGG: Brain lower grade glioma
LIHC: Liver hepatocellular carcinoma
LUAD: Lung adenocarcinoma
LUSC: Lung squamous cell carcinoma
MESO: Mesothelioma
OV: Ovarian serous cystadenocarcinoma
PAAD: Pancreatic adenocarcinoma
PCPG: Pheochromocytoma and paraganglioma
PRAD: Prostate adenocarcinoma
READ: Rectum adenocarcinoma
SARC: Sarcoma
SKCM: Skin cutaneous melanoma
STAD: Stomach adenocarcinoma
TGCT: Testicular germ cell tumors
THCA: Thyroid carcinoma
THYM: Thymoma
UCEC: Uterine corpus endometrial carcinoma
UCS: Uterine carcinosarcoma
